# Venous Wall of Patients with Chronic Venous Disease Exhibits a Glycolytic Phenotype

**DOI:** 10.3390/jpm12101642

**Published:** 2022-10-03

**Authors:** Oscar Fraile-Martinez, Cielo García-Montero, Miguel Ángel Alvarez-Mon, Ana M. Gomez-Lahoz, Jorge Monserrat, Maria Llavero-Valero, Fernando Ruiz-Grande, Santiago Coca, Melchor Alvarez-Mon, Julia Buján, Natalio García-Honduvilla, Jose V. Saz, Miguel A. Ortega

**Affiliations:** 1 Department of Medicine and Medical Specialities, Faculty of Medicine and Health Sciences, University of Alcalá, 28801 Alcalá de Henares, Spain; 2 Ramón y Cajal Institute of Sanitary Research (IRYCIS), 28034 Madrid, Spain; 3 Endocrinology Serive Hospital Universitario Infanta Leonor, 28031 Madrid, Spain; 4 Department of Vascular Surgery, Príncesa Hospital, 28834 Madrid, Spain; 5 Immune System Diseases-Rheumatology and Internal Medicine Service, University Hospital Príncipe de Asturias, CIBEREHD, 28806 Alcalá de Henares, Spain; 6 Department of Biomedicine and Biotechnology, Faculty of Medicine and Health Sciences, University of Alcalá, 28801 Alcalá de Henares, Spain

**Keywords:** chronic venous disease (CVeD), varicose veins (VVs), glycolytic phenotype, histopathological markers

## Abstract

Chronic venous disease (CVeD) is a rising medical condition characterized by a broad spectrum of disorders in the venous system. Varicose veins (VVs) represent a frequent clinical manifestation of CVeD, particularly in the lower limbs. Prior histopathological studies have defined a set of alterations observed in the venous wall of patients with VVs, affecting their structure and behavior. Metabolic changes in the veins appear to be a critical biological mechanism aiding our understanding of the pathogenesis of CVeD. In this sense, previous studies have identified a potential role of a glycolytic phenotype in the development of different vascular disorders; however, its precise role in CVeD remains to be fully explored. Thus, the aim of the present study was to analyze the gene and protein expression of glucose transporter 1 (GLUT-1) and the glycolytic enzymes PGK-1, ALD, GA3PDH and LDH in the VVs of patients with CVeD (*n* = 35) in comparison to those expressed in healthy subjects. Our results display enhanced gene and protein expression of GLUT-1, PGK-1, ALD, GA3PDH and LDH in patients with CVeD, suggesting a glycolytic switch of the venous tissue. Greater understanding of the impact of this glycolytic switch in patients with CVeD is required to define a possible pathophysiological role or therapeutic implications of these changes.

## 1. Introduction

Chronic venous disease (CVeD) comprises a broad spectrum of changes in the venous system commonly manifested in form of telangiectasias, reticular and varicose veins (VVs) [1]. Epidemiologically, the prevalence of CVeD is estimated to be about 15–80%, with such a range due to significant differences in study designs and target populations [2,3,4]. In the United States alone, there are 25 million people with CVeD and more than 6 million with advanced stages, with a total annual cost of more than $3 billion per year [5]. Women are more prone to suffer from CVeD due to different biological factors; according to the Framingham study, the annual incidence of CVeD is around a 2.6% for females and 1.9% for males [6,7]. Apart from the female sex, other important risk factors to suffer from CVeD include aging, sedentarism, obesity, pregnancy, smoking and family history [8,9,10].

Pathophysiologically, CVeD is characterized by an ambulatory venous hypertension, which affects the venous valves, compromising the venous return, frequently in the lower limbs [11,12]. The persistent hypertension is a major source of local and systemic inflammation, leading to an increase in oxidative stress, endothelial dysfunction, chronic hypoxia, matrix remodeling and so on [13,14,15,16]. Eventually, in advanced stages of the disease, skin changes such as pigmentation, lipodermatosclerosis and ulcerations can be observed [17]. Despite being a common malady, compelling evidence supports that this condition is frequently underdiagnosed and undertreated, generating incapacities to work and limiting the quality of life of the affected patients, especially in their most advanced presentations [18,19,20,21]. Thus, further efforts are needed in order to understand the onset and progression of this global concern, aiding in explaining the pathophysiological signature in the venous wall of these patients. 

Metabolic changes can be one of the putative biological mechanisms implicated in the pathogenesis of CVeD [22]. Sustained inflammation, hypoxia and endothelial injury are associated with a metabolic reprogramming of the vascular wall [23]. Both endothelial and vascular smooth muscle cells strongly depend on glucose metabolism and glycolysis in physiological conditions [23,24], and likewise, an aberrant regulation of these processes appears to be critically involved in vascular disorders [25,26]. Histological alterations in glycolysis markers in the placenta of women with CVeD has been recently demonstrated [27]. In addition, quantitative proteomic analysis has identified a set of glycolytic components significantly altered in VVs of patients with CVeD [28]. However, the precise status of the glucose metabolism in the venous tissue of patients with CVeD remains to be fully understood. In this sense, the aim of the present study is to describe gene and protein expression of the glucose transporter 1 (GLUT1), together with the glycolytic enzymes phosphoglycerate kinase 1 (PGK1), aldolase (ALD), glyceraldehyde-3-phosphate dehydrogenase (GA3PDH) and lactate dehydrogenase (LDH), in order to describe the glycolytic status in the VVs of subjects with CVeD.

## 2. Patients and Methods

### 2.1. Study Design 

In this cross-sectional study, we included the great saphenous vein wall from 35 patients with a clinical diagnosis of CVeD, with a median age of 47.00 years [27.00–68.00] after saphenectomy. Moreover, the great saphenous vein wall of 27 subjects with no history of CVeD (HV) after undergoing organ extraction surgery for bypass were also included. For HV patients, color flow duplex ultrasonography (CFDUS) studies were performed. This study was led following basic ethical principles (autonomy, harmlessness, beneficenc, and distributive justice) and according to the standards of Good Clinical Practice and the principles defined in the Declaration of Helsinki (2013) and the Oviedo Convention (1997). The collected data and information complied with the current legislation on data protection (Organic Law 3/5 December 2018 on the Protection of Personal Data and the Guarantee of Digital Rights and Regulation (EU) 2016/679). This project (FIS-PI18/00912) was approved by the Clinical Research Ethics Committee of the Gómez-Ulla-UAH Defence Hospital (37/17). Written informed consent was provided by all patients who were enrolled in this study.

The inclusion criteria were as follows: men and women diagnosed with CVeD; body mass index (BMI) ≤ 25 kg/m^2^; with and without venous reflux in the great saphenous vein; and commitment to follow-up during the pre- and postoperative periods as well as to provide tissue samples. The exclusion criteria were as follows: patients without access to their clinical history; patients with medical conditions affecting the cardiovascular system (infections, diabetes, hypertension and dyslipidemia); venous malformations or arterial insufficiency; toxicological habits; and those who were uncertain of participating in the continued monitoring. The clinical diagnosis of CVeD and the evaluation of venous reflux were based on a noninvasive color Doppler ultrasound (7.5–10 MHz) of the superficial and deep vein systems. The Classification System for Chronic Venous Disorders (CEAP), which is based on clinical, etiologic, anatomic and pathophysiologic data, was applied previously to venous extraction [29]. All patients included presented a CEAP clinical class (C1, *n* = 9; C2, *n* = 19; C3, *n* = 7), and all patients had primary disease (Ep). 

### 2.2. Sample Processing 

Fragments of the great saphenous vein in its middle portion from CVeD and HV were placed in two sterile tubes: one containing minimal essential medium (MEM) with 1% antibiotic/antimycotic (Thermo Fisher Scientific, Waltham, MA, USA) and the other with RNAlater solution (Ambion, Austin, TX, USA). Then, all samples were transported refrigerated within 4 h of being extracted to the Faculty of Medicine of the University of Alcalá, Department of Medicine and Medical Specialties, for further processing. In the laboratory, samples were processed in sterile conditions under a Telstar AV 30/70 Müller 220 Hz class II laminar flow hood (Group Telstar SA). The tubes preserved in MEM were destined for histological studies. First, these samples were washed/hydrated several times with MEM without antibiotics to remove blood cells ,and divided into fragments that were subsequently placed in F13 (60% ethanol, 20% methanol, 7% polyethylene glycol and 13% distilled H_2_O). Then, samples were dehydrated and embedded in paraffin blocks in agreement with established protocols [30]. The samples placed in RNAlater^®^ were stored in 1 mL of this solution at −80 °C until posterior processing for gene expression analysis.

### 2.3. Analysis of Gene Expression Using RT-qPCR

Gene expression was assessed by the use of real-time PCR (RT-qPCR) according to the following steps: First, RNA extraction was achieved by the guanidine–phenol–chloroform isothiocyanate method following the protocol published by Garcia-Honduvilla et al. [31]. Primers were designed by the Primer-BLAST tool [32] and the Auto-Dimer application [33]. A StepOnePlus™ system was used to perform quantitative PCR (qPCR), using the relative standard curve method. To do so, 5 μL of each sample, previously diluted in nuclease-free water, was mixed with 10 μL of the intercalating agent iQ™ SYBR^®^ Green Supermix (BioRad laboratories), 1 μL of forward primer, 1 μL of reverse primer and 3 μL of DNase- and RNase-free water in a 96-well MicroAmp^®^ plate (Applied Biosystems-Life Technologies, Waltham, MA, USA), with a final volume of 20 μL. The final results were normalized and compared with the expression of the constitutively expressed gene TBP (Table 1). Fluorescence detection was measured at the end of each amplification cycle and at each step of the dissociation curve. Then, the data obtained for each gene were extrapolated in a standard curve. In the plates, the samples were assayed in triplicate, the standard curve was assayed in duplicate and the remaining two wells were filled with negative controls.

### 2.4. Study of Protein Expression through Immunohistochemistry 

The antigen-antibody reaction was studied following the ABC (avidin–biotin complex) method with peroxidase as chromogen in agreement with established protocols [34]. First, samples were incubated at 4 °C overnight in a dilution composed of primary antibody, 3% BSA and PBS, as detailed in Table 2. Then, incubation with the secondary antibody bound to biotin and diluted in PBS was conducted for 90 min at room temperature (Table 3). Afterward, conjugated ExtrAvidin^®^-Peroxidase (Sigma-Aldrich, St. Louis, MO, USA) was applied for 60 min at room temperature (1:200 dilution in PBS), using the chromogenic substrate diaminobenzidine (Kit DAB, SK-4100, Vector Laboratories, Burlingame, CA, USA; prepared immediately before exposure as follows: 5 mL of distilled water, two drops of buffer, four drops of DAB, two drops of hydrogen peroxide). This procedure allows us to detect a brown staining. In every immunohistochemistry, sections from the same tissue were used as negative controls, in which the incubation with primary antibody was substituted with incubation in a blocking solution (PBS).

### 2.5. Histological Observation and Statistical Analysis 

Five sections and ten fields per section were randomly analyzed for each patient and HV classified in their established groups. Patients were considered positive when the marked average area was equal to or higher than 5% of the total, following the IRS score method [35]. The slides were then examined under a Zeiss Axiophot optical microscope (Carl Zeiss, Oberkochen, Germany) equipped with an AxioCam HRc digital camera. The statistical analysis was performed using the program GraphPad Prism^®^ 8.0. Mann–Whitney U tests were studied in this work. The obtained data are expressed as the median with standard deviation (SD), and significance was established at *p* < 0.05 (*), *p* < 0.01 (**) and *p* < 0.001 (***).

## 3. Results

### 3.1. CVeD Patients Show Increased Expression of GLUT-1 in the Venous Wall

**Figure 1:**
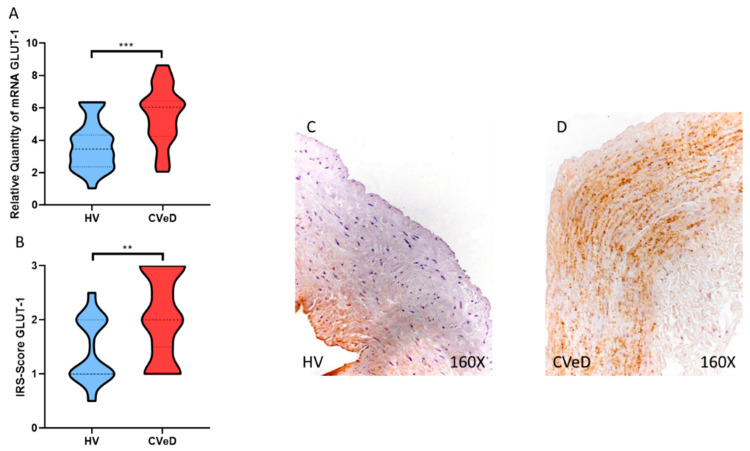
(**A**). GLUT-1 mRNA expression in the HV group (Healthy controls) and CVeD patients (chronic venous disorder). (**B**). IRS-Scores for GLUT-1 in the venous wall of the HV group and the CVeD group. (**C**,**D**). Images showing the immunostaining for GLUT-1 in the C group in the three tunicae of the venous wall. *p* < 0.01 (**) and *p* < 0.001 (***).

We found a significant increase in GLUT-1 gene expression in the venous wall of patients with CVeD by RT-qPCR (HV = 3704 ± 1500 vs. CVeD = 5576 ± 1752 RQ, *** *p* = 0.0001, Figure 1A). In this regard, we observed through immunohistochemical studies a significant increase in GLUT-1 protein expression in the venous wall of patients with CVeD (HV = 3704 ± 1500 vs. CVeD = 5576 ± 1752 IRS, ** *p* = 0.0013, Figure 1B–D).

### 3.2. CVeD Patients Show Increased Expression of Glycolytic Enzymes

**Figure 2:**
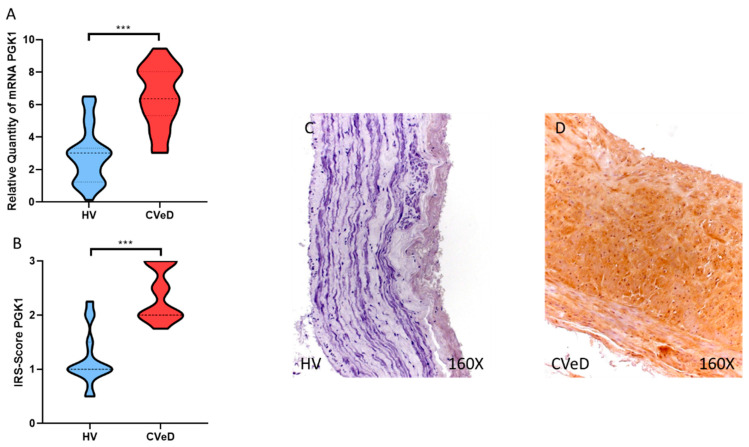
(**A**). PGK1 mRNA expression in the HV group (healthy controls) and CVeD patients (chronic venous disorder). (**B**). IRS-Scores for PGK1 in the venous wall of the HV group and the CVeD group. (**C**,**D**). Images showing the immunostaining for PGK1 in the C group in the three tunicae of the venous wall. *p* < 0.001 (***).

Our results showed a significant increase in PGK1 expression in the venous wall of CVeD patients by RT-qPCR (HV = 2879 ± 1731 vs. CVeD = 6479 ± 1819 RQ, *** *p* < 0.0001, Figure 2A). In addition, histopathological studies demonstrated a significant increase in PGK1 protein expression in the venous wall of CVeD patients (HV = 1.185 ± 0.503 vs. CVeD = 2.379 ± 0.475 IRS, *** *p* < 0.0001, Figure 2B–D). 

**Figure 3:**
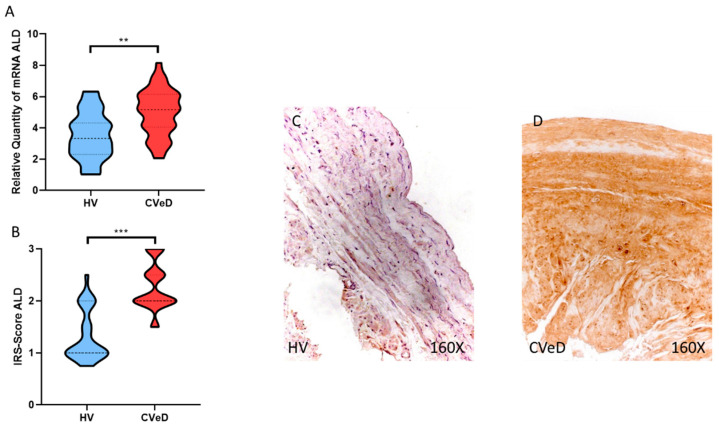
(**A**). ALD mRNA expression in the HV group (healthy controls) and CVeD patients (chronic venous disorder). (**B**). IRS-Scores for ALD in the venous wall of the HV group and the CVeD group. (**C**,**D**). Images showing the immunostaining for ALD in the C group in the three tunicae of the venous wall. *p* < 0.01 (**) and *p* < 0.001 (***).

In this regard, we observed a significant increase in ALD gene expression in the venous wall of CVeD patients (HV = 3,540 ± 1,509 vs. CVeD = 4,982 ± 1,486 RQ, ** *p* = 0.0017, Figure 3A). Immunohistochemical studies showed a significant increase in ALD protein expression in the venous wall of CVeD patients (HV = 1.324 ± 0.494 vs. CVeD = 2.271 ± 0.459 IRS, *** *p* < 0.0001, Figure 3B–D). 

**Figure 4:**
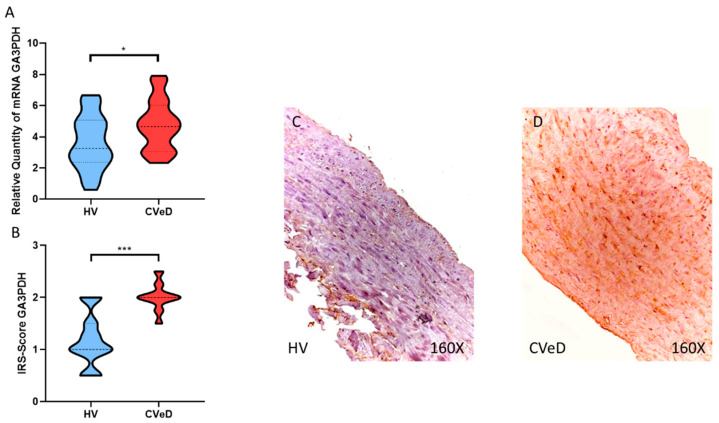
(**A**). GA3PDH mRNA expression in the HV group (Healthy controls) and CVeD patients (chronic venous disorder). (**B**). IRS-Scores for GA3PDH in the venous wall of the HV group and the CVeD group. (**C**,**D**). Images showing the immunostaining for GA3PDH in the C group in the three tunicae of the venous wall. *p* < 0.05 (*) and *p* < 0.001 (***).

**Figure 5:**
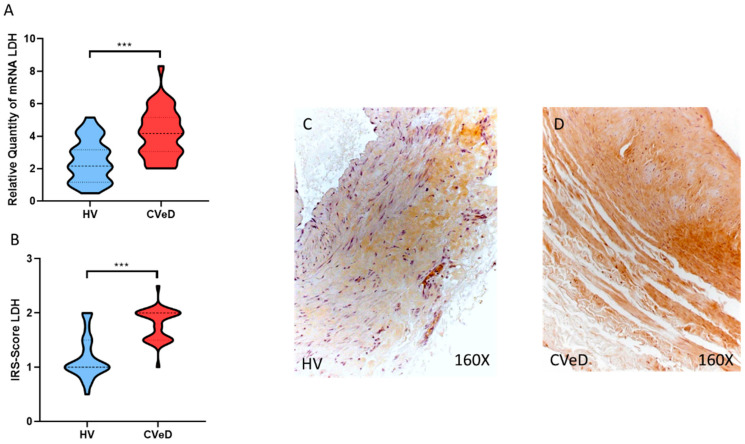
(**A**). LDH mRNA expression in the HV group (healthy controls) and CVeD patients (chronic venous disorder). (**B**). IRS-Scores for LDH in the venous wall of the HV group and the CVeD group. (**C**,**D**). Images showing the immunostaining for LDH in the C group in the three tunicae of the venous wall. *p* < 0.001 (***).

Another of the glycolytic enzymes that showed a significant increase was GA3PDH expression in the venous wall of CVeD patients by RT-qPCR (HV = 3.617 ± 1.731 vs. CVeD = 4.7609 ± 1.598 RQ, * *p* = 0.0201, Figure 4A). In addition, immunohistochemical studies showed a significant increase in GA3PDH protein expression in the venous wall of CVeD patients (HV = 1.185 ± 0.493 vs. CVeD = 2.014 ± 0.257 IRS, *** *p* < 0.0001, Figure 4B–D). Finally, we observed a significant increase in LDH gene expression in the venous wall of CVeD patients (HV = 2512 ± 1293 vs. CVeD = 4171 ± 1497 RQ, *** *p* < 0.0001, Figure 5A). In parallel, we observed a significant increase in LDH protein expression in the venous wall of CVeD patients (HV = 1.167 ± 0.398 vs. CVeD = 1.793 ± 0.294 IRS, *** *p* < 0.0001, Figure 5B–D).

## 4. Discussion

In the present work, we have demonstrated the existence of a glycolytic switch measured by upregulated expression of GLUT-1, PGK-1, ALD, GA3PDH and LDH in the venous wall of patients with CVeD. This research adds further knowledge on the great variety of histopathological changes occurring in the VVs, opening up potential lines of research to achieve a deeper understanding of CVeD. 

Glycolysis appears to be the primary energy-sustaining process in endothelial cells; however, under pathological conditions, the glycolytic and other metabolic processes are hyperactivated, leading to enhanced oxidative stress, cell dysfunction and eventually cell death [36]. Glycolysis is a process closely related to a hypoxic environment [37]. Persistent hypoxia is a major feature of CVeD pathogenesis, leading to prominent changes in the venous wall, especially affecting the vascular endothelium and smooth muscle cells [17]. From a molecular perspective, this hypoxic environment leads to the activation of the hypoxia-inducible factor (HIF), a major transcription factor that regulates a set of genes and their products [38]. Increased activation of HIF-1 alpha has been reported in patients with VVs, especially in those with venous reflux and advanced stages [39,40]. In this sense, previous studies have demonstrated that hypoxia can induce lactate secretion and glycolytic efflux in the venous tissue [41]. In turn, it is well-established that a glycolytic phenotype is required for the process of angiogenesis [42]. Altered markers of angiogenesis have been described in the VV of patients with CVeD as a compensatory mechanism of the hypoxic environment [43]. Thus, the glycolytic switch observed in the VV may be a consequence of the persistent hypoxia, being involved in the altered angiogenesis reported in these structures. 

In this study, we observed increased GLUT-1 expression in patients with CVeD. GLUT-1 is involved in the transport of glucose galactose, mannose, glucosamine and ascorbic acid, being ubiquitously distributed in the different tissues, regulating multiple physiological and pathological processes [44]. Smooth muscle cells can be especially sensitive to those changes occurring in GLUT-1 expression, as previous studies have reported that augmented GLUT-1 expression in these cells alters vascular contractility and promotes inflammation and medial hypertrophy, which are associated with upregulated transforming growth factor-β (TGF-β) activity [45]. Aberrant expression of TGF-β can be observed in the VV of patients with CVeD, especially in the media layer’s advanced stages ([46,47]). Augmented levels of GLUT-1 and glycolytic activity in the smooth muscle cells may be related to abnormal behavior of these cells, aiding our understanding of the progression of CVeD. 

Likewise, we have also demonstrated that patients with CVeD display enhanced expression of the glycolytic enzymes (PGK-1, ALD, GA3PDH and LDH). PGK-1, together with ALD, are tightly regulated by the phosphatidyl inositol 3 kinase (PI3K)/Akt pathway, which is crucial for enhancing proliferation and different cellular mechanisms [48,49]. The central role of this pathway has been previously defined in the pathogenesis of CVeD, being directly associated with accelerated aging of the venous tissue and HIF expression [40]. Upstream, an altered PAPP-A/IGF-1/STC-2 axis has been observed in the venous wall of VVs, influencing in the PI3K/Akt pathway toward hyperactivation [50]. Thus, tissue hypoxia and PI3K/Akt pathway hyperactivation may be responsible for the upregulation of these enzymes in patients with CVeD, influencing the glycolytic phenotype.

GA3PDH seems to influence not only glycolytic steps but also several proteins, modulating their function and affecting cells’ fate [51]. The role of this enzyme in vascular pathologies has been explored in previous works [52]. Increased GA3PDH activity was found to be responsible for promoting the activation of NF-κB, enhancing the transcription and activity of HIF and promoting tissue vascularization [53]. Finally, LDH expression seems to indicate an increase in the production of lactate in the venous wall of patients with VVs. Lactate is a major signaling molecule, with modulatory roles in the inflammatory response, immune system, metabolism, angiogenesis and even as a potential epigenetic regulator [54]. In vascular smooth muscle cells, lactate induces a synthetic phenotype, suggesting a potential pathophysiological role of this molecule in cardiovascular diseases [55]. In the endothelium, lactate drives increased production of vascular endothelial growth factor (VEGF) and modulates angiogenesis [56]. Increased VEGF levels have been reported in the vascular wall of patients with VVs [57], and lactate could be responsible for this. Likewise, prior research works have also described altered expression of LDH in the varicose venous wall [58], suggesting a possible but still inconclusive role of the glycolytic phenotype in CVeD. 

## 5. Conclusions

In the present work, we have demonstrated increased gene and protein expression of the glucose transporter GLUT-1 and the glycolytic enzymes PGK-1, ALD, GA3PDH and LDH in the VVs of patients with CVeD. Collectively, these data support a glycolytic phenotype detected in the pathological veins, possibly aiding in explaining the altered behavior, structure and pathophysiological changes reported in the venous tissue. This study, as such, opens up novel lines of research to be explored in these patients. However, it is also true that our study has some important limitations. For instance, we did not perform other complementary techniques to study protein expression levels such as a Western blot, nor did we distinguish the proximal and distal segments of the great saphenous vein. In this sense, it is necessary to expand the cohorts and carry out a broader stratification by degrees of disease. Nonetheless, this is valuably the first histopathological study to have reported the importance of these markers in this highly prevalent disease.

## Figures and Tables

**Table 1:** Primer sequences used in RT-qPCR and temperature (Tm). The data for all the experiments are listed in Supplementary Materials.

Gene	Sequence Fwd (5′ → 3′)	Sequence Rev (5′ → 3′)	Tm
TBP	TGCACAGGAGCCAAGAGTGAA	CACATCACAGCTCCCCACCA	60 °C
GLUT-1	GGCCGGTAAGTAGGAGAGGT	ATTGAATTCCGCCTGGGGAC	61 °C
PGK1	TGGACTGTGGTCCTGAAAGC	GTTCTCCATTCCACCTTCCTCT	58 °C
ALD	CCAGAAGGGTCCAGCTTCAA	CAACACCGCCCTTGGATTTG	58 °C
GA3PDH	GGA AGG TGA AGG TCG GAG TCA	GTC ATT GAT GGC AAC AAT ATC CAC T	60 °C
LDH	CAGGTGGTTGAGAGGGTCTT	AGGGTTGCCCAAGAATAGCC	59 °C

**Table 2:** Different antibodies (A. primary, B. secondary) used in the immunohistochemistry studies performed, with dilutions and protocol features. The data for all the experiments are listed in Supplementary Materials.

Antigen	Species	Dilution	Provider	Protocol Specifications
**PGK1**	Rabbit	1:500	Abcam (ab154613)	10 mM Sodium citrate pH = 6 before incubation with the blocking solution.
**GLUT1**	Mouse	1: 200	Abcam (ab40084)	10 mM Sodium citrate pH = 6 before incubation with the blocking solution.
**ALD**	Rabbit	1:250	Abcam (ab252953)	0.1% Triton with PBS, 10 min, before incubation with the blocking solution
**GA3PDH**	Rabbit	1:500	Abcam (ab 134187)	0.1% Triton with PBS, 10 min, before incubation with the blocking solution
**LDH**	Rabbit	1:750	Abcam (ab 199553)	0.1% Triton with PBS, 10 min, before incubation with the blocking solution

**Table 3:** Different antibodies (A. primary, B. secondary) used in the immunohistochemistry studies performed, with dilutions and protocol features.

Antigen	Species	Dilution	Provider	Protocol Specifications
**IgG (Mouse)**	Goat	1:300	Sigma (F2012/045K6072)	
**IgG (Rabbit)**	Mouse	1: 1000	Sigma (RG-96/B5283)	

## Data Availability

The data used to support the findings of the present study are available from the corresponding author on request.

## Supplementary Materials

The data for all the experiments. The following are available online at https://www.mdpi.com/article/10.3390/jpm12101642/s1.
